# Chikungunya Manifestations and Viremia in Patients Who Presented to the Fever Clinic at Bangkok Hospital for Tropical Diseases during the 2019 Outbreak in Thailand

**DOI:** 10.3390/tropicalmed6010012

**Published:** 2021-01-21

**Authors:** Hisham A Imad, Juthamas Phadungsombat, Emi E Nakayama, Sajikapon Kludkleeb, Wasin Matsee, Thitiya Ponam, Keita Suzuki, Pornsawan Leaungwutiwong, Watcharapong Piyaphanee, Weerapong Phumratanaprapin, Tatsuo Shioda

**Affiliations:** 1 Mahidol-Osaka Center for Infectious Diseases, Faculty of Tropical Medicine, Mahidol University, Bangkok 10400, Thailand; juthamasps@gmail.com (J.P.); emien@biken.osaka-u.ac.jp (E.E.N.); shioda@biken.osaka-u.ac.jp (T.S.); 2 Department of Viral Infections, Research Institute for Microbial Diseases, Osaka University, Suita, Osaka 565-0871, Japan; keita-s@ml.tanaka.co.jp; 3 Bangkok Hospital for Tropical Diseases, Faculty of Tropical Medicine, Mahidol University, Bangkok 10400, Thailand; sajikapon.klu@mahidol.ac.th (S.K.); wasin.mat@mahidol.edu (W.M.); thitiya.pon@mahidol.ac.th (T.P.); watcharapong.piy@mahidol.ac.th (W.P.); weerapong.phu@mahidol.ac.th (W.P.); 4 POCT Products Business Unit, TANAKA Kikinzoku Kogyo, Hiratsuka 254-0076, Japan; 5 Tropical Medicine Diagnostic Reference Laboratory, Faculty of Tropical Medicine, Mahidol University, Bangkok 10400, Thailand; pornsawan.lea@mahidol.ac.th

**Keywords:** *Alphavirus*, chikungunya virus, East Central South African lineage, Indian Ocean sub-lineage, acute febrile illness, viremia, arthritides

## Abstract

Chikungunya virus is an *Alphavirus* belonging to the family *Togaviridae* that is transmitted to humans by an infected *Aedes* mosquito. Patients develop fever, inflammatory arthritis, and rash during the acute stage of infection. Although the illness is self-limiting, atypical and severe cases are not uncommon, and 60% may develop chronic symptoms that persist for months or even for longer durations. Having a distinct periodical epidemiologic outbreak pattern, chikungunya virus reappeared in Thailand in December 2018. Here, we describe a cohort of acute chikungunya patients who had presented to the Bangkok Hospital for Tropical Diseases during October 2019. Infection was detected by a novel antigen kit and subsequently confirmed by real-time RT-PCR using serum collected at presentation to the Fever Clinic. Other possible acute febrile illnesses such as influenza, dengue, and malaria were excluded. We explored the sequence of clinical manifestations at presentation during the acute phase and associated the viral load with the clinical findings. Most of the patients were healthy individuals in their forties. Fever and arthralgia were the predominant clinical manifestations found in this patient cohort, with a small proportion of patients with systemic symptoms. Higher viral loads were associated with arthralgia, and arthralgia with the involvement of the large joints was more common in female patients.

## 1. Introduction

Several arboviral infections are endemic to Thailand [1,2,3]. Amongst these, chikungunya virus (CHIKV) has the potential for re-emerging and is notorious for its morbidity [4]. *Chikungunya* is an *Alphavirus* belonging to the family *Togaviridae* that is transmitted by an infected *Aedes* mosquito [5,6]. There are several lineages of the chikungunya virus. These include the Asian Urban lineage (AUL), which is historically constrained to Southeast Asia. Recently, genomic polymorphism in this lineage has led to a sub-lineage, the Asian/American lineage that now circulates in South America [7]. The East, Central, and South African lineage (ECSA) re-emerged in Thailand two decades ago and has been mainly restricted to epidemics in the African continent and South and Southeastern Asia [8]. In the year 2004, the ECSA caused an outbreak in Kenya, which subsequently spread to the islands in the Indian Ocean, which led to the emergence of a mutated form known as the Indian Ocean lineage (IOL) that now circulates in the Indian subcontinent and Europe [9]. The West African lineage remains entirely isolated to West Africa [10]. The virus was first isolated in Thailand four decades ago and is known to have an epidemiological distinct periodic outbreak worldwide [11]. Thailand has seen several outbreaks of chikungunya disease over the years [12,13,14,15,16,17,18,19,20,21,22]. Many reports exist of the virus being exported out of the country to non-endemic regions by returning viremic travelers [23,24,25,26]. The two major transmission cycles of the virus are the sylvatic cycle and the urban cycle. In the urban transmission cycle, infected humans fuel the outbreak as they amplify the virus for circulating *Aedes* mosquitos to effectively transmit the virus to others [27]. After an incubation period of 2–10 days, most patients develop an abrupt onset high-grade fever associated with typical characteristics of chikungunya infection, like arthralgia, myalgia, and rash [28,29,30,31,32,33,34,35]. The majority of patients recover after a spontaneous self-limiting illness. A subset of individuals exhibit atypical manifestations, and some develop a chronic course of illness [36,37,38]. Based on clinical and laboratory profiles, it is difficult for clinicians to distinguish chikungunya infection from other circulating pathogens such as dengue and Zika viruses [39,40]. To overcome this obstacle, researchers at the Mahidol-Osaka Center for Infectious Diseases at Mahidol University in Thailand, in collaboration with the Department of Viral Infections at Osaka University in Japan, have developed a novel prototype rapid point-of-care immunochromatography test kit that identifies suspected chikungunya cases based on detection of the E1 antigen [41,42]. This prototype’s performance and updated test kits have been validated using clinical specimens from different parts of the world [43,44]. This evaluation proved that our test kits have a 92% sensitivity (95% confidence interval 85.0–95.9) and a 100% specificity. The specificity of the test kit was assessed using 100 dengue-positive sera [43]. This new diagnostic breakthrough in translational research is promising for easy and quick detection of suspected cases of chikungunya infections during outbreaks. As of December 2019, Thailand was facing one of the largest recorded chikungunya outbreaks [21]. According to the Thai Ministry of Public Health, in 2018, there were 3570 cases reported, and 8774 new cases were reported up to the end of October 2019 [45]. We supported the Fever Clinic at the Bangkok Hospital for Tropical Diseases by utilizing this immunochromatographic kit to detect cases of acute chikungunya in patients who had presented during the surge of the epidemic in October 2019. Infection was confirmed by real time RT-PCR using serum collected at presentation. Other possible acute febrile illness-causing pathogens such as influenza, dengue, and malaria were excluded using serology and blood smear for parasites. We explored the sequence of clinical manifestations, including the hematological profile, that occurred during the presentation to the Fever Clinic and evaluated the association of viral load with clinical and laboratory parameters to know chikungunya-specific features.

## 2. Materials and Methods

### 2.1. Patients

During October 2019, while the chikungunya outbreak surged in central Thailand and cases peaked in Bangkok, the Mahidol-Osaka Center for Infectious Diseases at the Faculty of Tropical Medicine, Mahidol University provided diagnostic support to the Fever Clinic at Bangkok Hospital for Tropical Diseases. Twenty-six patients who were clinically suspected of having chikungunya infection were diagnosed using a prototype lateral flow immunochromatography rapid point-of-care test kit, and this was subsequently confirmed with real-time RT-PCR. 

### 2.2. Chikungunya Antigen Testing by Immunochromatography

The prototype kit was developed at Mahidol-Osaka Center for Infectious Diseases, and the details of the immunochromatography kit have been described previously, including the validation of the kit performance [41,42,43]. Briefly, left-over serum from routine investigation performed at the Fever Clinic was obtained from the hospital central laboratory. Thirty microliters of serum were used to detect the antigen by mixing with 60 μL of extraction buffer in a microtube. Next, the immunochromatography strip was placed in the mixed solution of serum and buffer. Results were interpreted by the appearance of the control and test bands assessed after 15 min.

### 2.3. Reverse Transcription Polymerase Chain Reaction

Quantification of chikungunya viral genomes was performed using methods previously described [46]. In brief, the RNA was extracted using a viral RNA extraction kit (QiAmp Viral RNA mini kit, Qiagen, Hilden, Germany). SYBR green quantitative RT-PCR was used to detect CHIKV by targeting the 120-bp region corresponding to E1, one of the envelope glycoproteins. The primer sequences were 5′-CTCATACCGCATCCGCATCAG-3′ (forward) and 5′-ACATTGGCCCCACAATGAATTTG-3′ (reverse). A standard curve was drawn using CHIKV RNA that was prepared from a CHIKV isolate obtained from an earlier study. The standard curve was prepared from six dilutions containing 10^1^ to 10^6^ PFU/mL, and the detection limit was determined to be about 10^2^ PFU/mL.

### 2.4. Structural Polyprotein Region Sequencing and Phylogenetic Analysis

To determine the nucleotide sequence of the structural polyprotein of CHIKV, total extracted RNA from real-time RT-PCR-positive samples was converted into cDNA using the superscript III first-strand synthesis system (Invitrogen, Carlsbad, CA, USA). Briefly, 4 µL RNA were mixed with a specific primer [47], dNTP, buffer, MgCl_2_, and DTT following the manufacturer’s protocol, and then 2 µL of 3′ half cDNA was further amplified with Primestar GXL DNA polymerase (Takara, Japan) using 3 primer sets [47,48], chf18/chr24, chf20/chr24, and chf23/3RT, to obtain 3 overlapping amplified products of 2.7 kb, 1.9 kb, and 2.0 kb. The amplicons were purified, cleaned (Nucleospin, MACHEREY-NAGEL, Germany), and sequenced (Macrogen, Seoul, Korea) using the primers chr20, chr21, chr22, chf21, chf22, chf24, and chf25 [48]. The obtained sequences were aligned to the reference CHIKV genotype African strain S27 (NC_004162.2) in AliView V1.26 [49], and the consensus sequence of the entire structural polyprotein region (3747 bp) was manually extracted and deposited in GenBank (Accession number LC598202-LC598210). The newly obtained sequences were aligned with the previously published sequences [45] and other public ECSA genotype in GenBank. The maximum likelihood tree of the dataset was constructed using IQTREE under TN+F+G4 and 1000 replicated of ultrafast bootstrap [50].

### 2.5. Clinical Data Analysis

Clinical and laboratory data were obtained from patient medical charts, and the data were analyzed retrospectively. For closer scrutiny of the clinical characteristics and manifestations occurring in the early phases during acute chikungunya infection, we categorized our cohort into two groups based on how early they presented to the hospital after developing symptoms. In our analysis, we considered patients presenting on days 1 to 2 after developing symptoms as “Group A”, and those who presented within 3 to 4 days as “Group B”. The two groups were further categorized based on the viral load to look for any association between viral load and clinical and laboratory parameters. For this analysis, we categorized the viral load to ≥10,000 copies and less than 10,000 copies as previously described [51]. Although there were three patients with underlying comorbidities, with the lack of any available metabolic data, we proceeded to include all 26 patients our analysis. Distributive frequencies of clinical manifestations were estimated in both groups. All categorical variables were tested for observed frequencies by the Pearson Chi-squared test. Wilcoxon Mann-Whitney was used for nonparametric testing. Pearson correlation analysis was performed to test for correlations with the hematological profile.

## 3. Results

**Figure 1:**
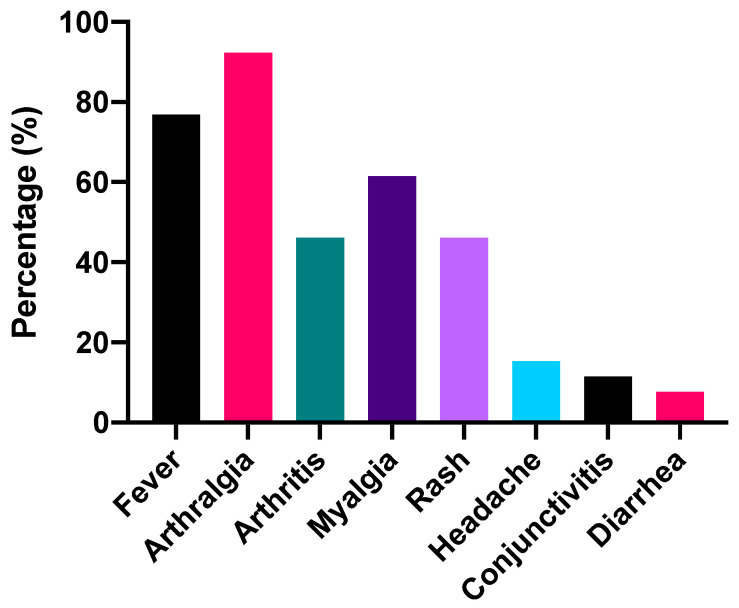
Frequencies of clinical manifestations at the time of presentation (*n* = 26).

During October 2019, 26 patients who presented to the Fever Clinic with clinically compatible features of chikungunya infection were analyzed in this report. There were 14 females and 12 males with median ages of 48.5 and 45 years, respectively. The demographic data are provided in Supplementary Table S1. Comorbidities such as diabetes mellitus and hypertension were only present in 3 patients. Twenty-four patients were of Thai nationality, and the remaining were foreigners. The symptoms at presentation are depicted in Figure 1. These include fever (76.9%), arthralgia (92.3%), arthritis (46.2%), myalgia (61.5%), rash (46.2%), headache (15.4%), conjunctivitis (11.5%), and diarrhea (7.7%). The overall median body temperature recorded at presentation was 38 °C with an interquartile range (IQR) of 37.7–39 °C.

### 3.1. Analysis of Clinical and Hematological Profiles

Thirteen patients presented to the Fever Clinic within 1 to 2 days following the onset of symptoms (Group A), and another 13 patients presented on the 3rd and 4th day of illness (Group B). Our analysis did not show any significant differences in the clinical findings in these two groups, as shown in Supplementary Table S2. We explored into these groups based on the gender. The clinical and laboratory features are provided in Table 1. In Group A, fever was reported by all male patients but not in all female patients. Arthralgia (*p* = 0.052) and involvement with large joints (*p* = 0.052) were significantly more common in females than males in Group A. Arthralgia was predominant in both sexes, in contrast to arthritis which appears to have been less common in Group A than B among males. Involvement of the small joints or both small and large joints occurred in more than half of the patients in both Groups A and B. Myalgia, rash, headache, conjunctivitis, and diarrhea occurred less frequently in this cohort. None of the patients had any bleeding manifestations at presentation. Interpreting the hematological profile of Group A, leukocytes were in the lower end of the normal reference range in both sexes. Amongst females, the median leukocyte count was 5050/μL, and in males 5100/μL. In Group B, the median leukocyte count was 3400/μL in females and 4900/μL in males. This difference in leukocytes between females and males was statistically significant (*p* = 0.010). The neutrophil was observed to increase in CHIKV patients, although median values were still within the normal range in both sexes in both groups. The median hematocrit percentage of 39.4% in females was slightly lower than the 42% in males in Group A. The hematocrit percentage was observed to be lower in females of Groups A and B compared to males. The median platelet counts were within the normal reference range in Group A.

### 3.2. Viral Load Analysis

We also determined if there was an association between the viral load and the clinical manifestation and laboratory profile. Our observations revealed that patients with a viral load of >10,000 copies/mL had a higher fever than patients with a viral load <10,000 copies/mL. This was statistically significant (*p* = 0.004). Similarly, patients with a higher viral load of 10,000 copies/mL manifested more arthralgia than patients with a viral load <10,000 copies/mL (*p* = 0.043). Other observations based on viral loads included those with a viral load >10,000 copies/mL tending to have an increase in manifestations such as the involvement of small joints, both joint types, arthritis, myalgia, and rash (Table 2). An analysis of the hematological profile based on the degree of viremia showed that those with a higher viral load of >10,000 copies/mL had a decreased lymphocyte count, with a median of 671 and IQR range of 440–812 compared with a lymphocyte percentage median (IQR) of 1323 (996–1508) when the viral load was <10,000 copies/mL (*p* = 0.000). Further, the neutrophil count was elevated to 3469 (2727–4797) when viral load was >10,000 copies/mL compared with a neutrophil count of 1656 (1155–3121) when the viral load was <10,000 copies/mL (*p* = 0.010). In addition to this, hematocrit, a parameter reflecting anemia, was lower 41.2 (38.2–43.5)) when the viral load was >10,000 copies/mL compared to that 44.6 (40.5–47.4)) (*p* = 0.055) when the viral load was <10,000 copies/mL. The actual distribution of the lymphocytes, neutrophils counts and the levels of hematocrit in relation to the viremia levels, are shown in Supplementary Figure S1. There were no statistically significant differences observed amongst the rest of the parameters included in the hematological profile between high and low viral loads.

### 3.3. The Absence of E1-A226V in Thailand 2019 CHIKV Strains 

**Figure 2:**
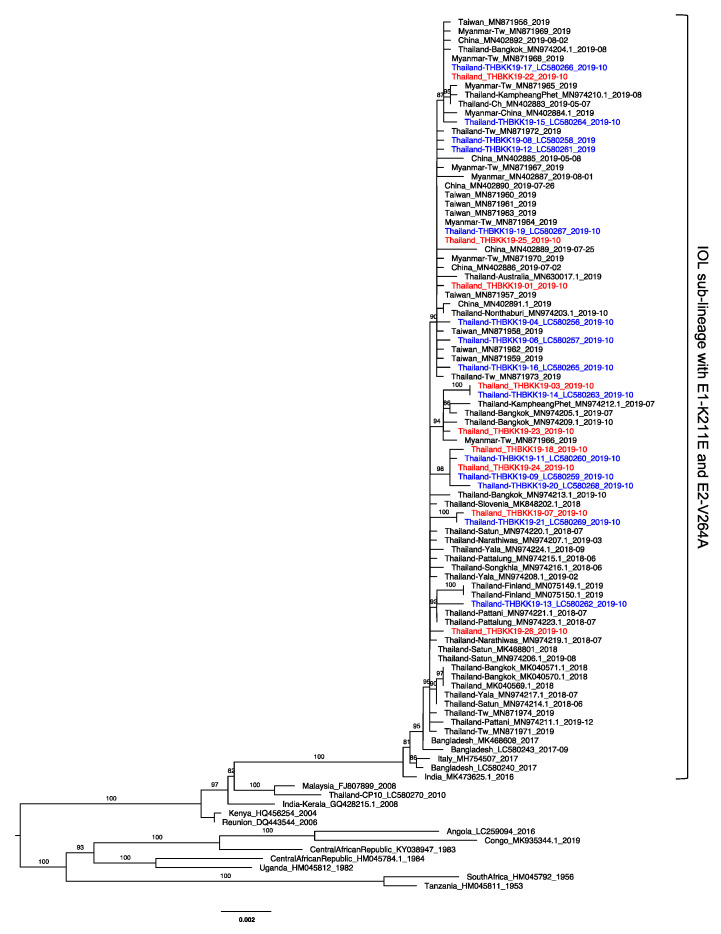
Phylogenetic tree based on the structural polyprotein region (3747 bp) of East/Central/South African chikungunya virus. A maximum likelihood tree was constructed under TN+F+G4 and 1000 ultrafast bootstrap replicates. The bootstrap values (%) over 80 are labeled on each branch. The 9 Bangkok CHIKVs sequenced in the present study and 14 sequenced in the previous study are indicated in red and blue, respectively. Bangkok CHIKVs were clustered with recent CHIKVs reported in Thailand and nearby areas. The IOL sub-lineage with E1-K211E and E2-V264A is indicated to the right.

Fourteen nearly whole CHIKV genomes from infected patients were sequenced and the genotypes determined in a previous study [52]. We were able to amplify the CHIKV structural protein gene regions from the sera of an additional 9 patients, though we failed to do so in the remaining 3 patients, probably due to low amounts of the virus within the sera (Supplementary Table S3). The structural protein regions in the amplified fragment were directly sequenced. The nucleotide sequences revealed our Thailand 2019 strains lacked an adaptive mutation at E1-A226V that was remarkably identified in the previous outbreak in Indian Ocean, Indian subcontinent, and Southeast Asia in 2005 and later on. Instead of E1-A226V, the all of them carried E1-226A (Supplementary Table S4). The phylogenetic tree showed all of them were the new IOL sub-lineage of ECSA CHIKV, closely related to the Bangladesh CHIKV strains of 2017 harboring E1-K211E and E2-V264A. The virus clustered into the previous 14 strains and other Thailand strains in the same clade of C2.3c [52], which is related to recent CHIKVs detected in Myanmar, China, and Taiwan in 2018–2019 (Figure 2). The E1-K211E and E2-V264A mutations were present in this clade. Moreover, all strains exhibited a common specific polymorphic mutation of K73R in the capsid protein (Supplementary Table S4).

### 3.4. Correlation Analysis

**Figure 3:**
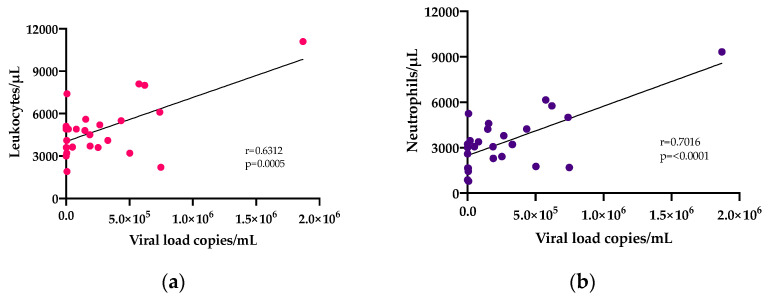
Correlation analysis of viral load with white blood cells. (**a**) Leukocytes vs. viremia, (**b**) Neutrophils vs. viremia.

**Figure 4:**
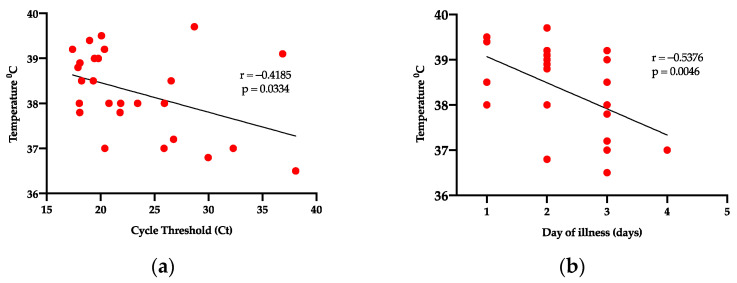
Correlation analysis of temperature against cycle threshold and day of illness. (**a**) Body temperature vs. cycle threshold. (**b**) Body temperature vs. day of illness.

**Figure 5:**
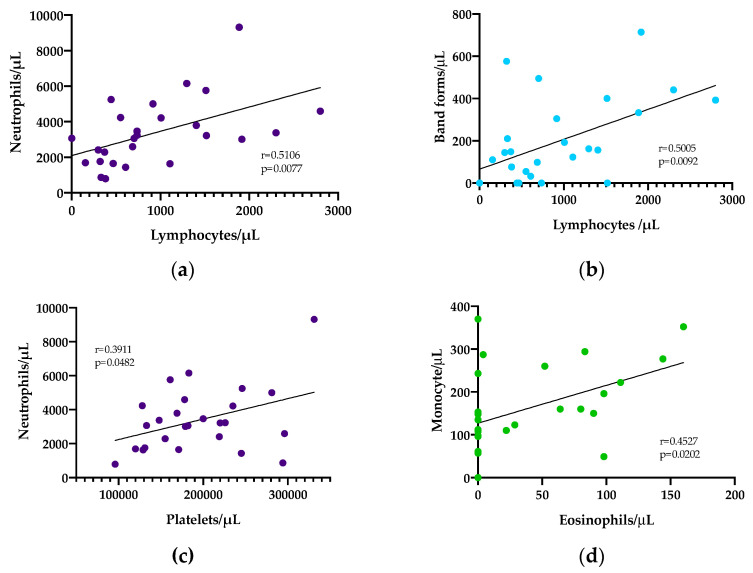
Correlation analysis of lymphocytes and neutrophils with other blood cell indices. (**a**) Lymphocytes vs. neutrophils, (**b**) Lymphocytes vs. band forms, (**c**) Neutrophils vs. platelets, and (**d**) Monocytes vs. eosinophils.

We also examined correlations between the hematological profile parameters, viral load, cycle threshold, day of illness, and the temperature recorded at presentation. Correlation analysis of the viral load showed a positive correlation between viremia and leukocyte count (r = 0.6612, *p* = 0.0005), including a positive correlation between viremia and neutrophil percentage (r = 0.7016, *p* = <0.0001) as shown in Figure 3. The temperature negatively correlated with the day of illness (r= −0.5376, *p* = 0.0046), including a negative correlation with the cycle threshold (r= −0.4185, *p* = 0.0334), as shown in Figure 4. The leukocyte counts positively correlated with neutrophils (r = 0.9612, *p* = <0.0001), lymphocytes (r = 0.5177, *p* = 0.0068) and platelets (r = 0.4432, *p* = 0.0233), as shown in Supplementary Figure S2. The cycle threshold positively correlated with the hematocrit percentage (r = 0.4661, *p* = 0.0164), hemoglobin (r = 0.4253, *p* = 0.0303), and negatively correlated with neutrophils (r= −0.4.117, *p* = 0.0367), as shown in Supplementary Figure S3. Lymphocytes positively correlated with neutrophils (r = 0.5106, *p* = 0.0077) and eosinophils (r = 0.5005, *p* = 0.0092). Neutrophils positively correlated with platelets (r= 0.3911, *p* = 0.0482), and monocytes positively correlated with eosinophils (r = 0.4527, *p* = 0.0202), and as shown in Figure 5.

## 4. Discussion

Chikungunya virus has re-emerged in many parts of the world and is considered to be a continuous global threat [53,54,55,56,57,58,59,60]. The reason for this resurgence is not fully elucidated, but is believed to be multifactorial, such as increased vector susceptibility and perhaps climate change [6,61,62]. Though the illness is self-limiting, infection results in increased morbidity and atypical manifestation, often with increased severity and fatalities [36,37,63,64,65,66,67,68,69,70]. Chikungunya virus causes similar clinical manifestations during the acute phase that could mimic other arboviruses, and there are no widely available point-of-care rapid diagnostics [43,71,72]. For these reasons, clinicians working in endemic regions and those treating returning travelers are challenged with diagnosing chikungunya infection [40,73,74,75].

The present outbreak in Thailand started in October 2018 [45]. Since then, the number of cases rapidly increased across the country and surged in Bangkok [21]. Based on phylogenetic analysis, the Thailand 2019 CHIKV strains even characterized to genotype ECSA-IOL lack E1-A226V, the adaptive mutation facilitating transmission in *Ae. albopictus* which observed in the IOL outbreak in 2005 [76]. The recent CHIKVs circulating 2016–present, particularly in India, Pakistan, Bangladesh, Italy, and Thailand were classified as the new IOL sub-lineage of genotype ECSA with specific amino acid mutations of E1-K211E and E2-V264A in the background of E1-226A [52]. The presence of both these mutations facilitated the fitness in *Ae. aegypti* [77]. Moreover, in 2017 in Italy, the E1-K211E and E2-V264A CHIKV variant was detected in *Ae. albopictus* and showed similar infection rate and transmission rate to E1-A226V CHIKV variant [78] whereas this new variant was detected in field-caught *Ae. aegypti* in Thailand [79]. These results suggested that this new sub-lineage IOL CHIKV had potential transmission capability either in temperate or tropical areas. Investigation of the vector competence are required. In addition, we further investigated the 23 CHIKVs collected in October 2019 were closely related to CHIKVs from southern Thailand collected earlier in late 2018 and recent circulating strains in 2019 from Myanmar, China, and Taiwan. We identified 3 clusters of closely related strains within Bangkok with residence proximity (Supplementary Figure S4).

The Bangkok Hospital for Tropical Diseases is a specialized hospital under the Faculty of Tropical Medicine administration at Mahidol University, where acute febrile undifferentiated infections are referred to [3]. We supported the Fever Clinic at the hospital with our rapid chikungunya virus diagnostic kits to help identify chikungunya cases during this outbreak [41,42]. We received clinical specimens regularly during the one-month period when the attending doctor at the Fever Clinic had ruled out dengue, malaria, and influenza. A total of 26 specimens were received, immunochromatography was positive for all of them, and the identification was subsequently confirmed by real time RT-PCR. Utilization of our antigen-based rapid point-of-care test kits allowed the attending doctor to diagnose and manage these patients in a timely manner. As most of the patients presented within 4 days after the onset of symptoms, our kits played a pivotal role in antigen detection, identifying chikungunya cases. This rapid detection of cases would have been impossible if diagnosis required using commercially available rapid point-of-care kits, which detect the antibody and work best after 5 days or more following the onset of illness [80].

Without laboratory-based diagnostics, chikungunya infection is difficult to distinguish from other acute febrile illnesses caused by common arboviruses like dengue viruses or Zika virus. Previous reports have described that chikungunya cases have higher viremia than dengue and Zika [40]. In contrast, others have reported that dengue cases have lower leucopenia and thrombocytopenia compared to cases of chikungunya or Zika [81]. We also compared the hematological profiles based on the day of illness between chikungunya patients in this study and our dengue cohort in a previous study [1], which might be useful in distinguishing cases of chikungunya based on the clinical manifestation in combination with hematological profile trends with respect to the day of illness (Supplementary Table S5). We observed that leucopenia and thrombocytopenia were frequently observed in dengue patients than in this cohort of chikungunya patients. A significant reduction in the neutrophil and platelet counts was present on the 3rd and 4th day of illness in dengue compared to chikungunya patients. Further, headache was more commonly reported in dengue patients during the 2nd day of illness. Headache, fever, and bleeding were more common during the 3rd and 4th day of illness in dengue patients. Bleeding in chikungunya cases has been reported to be rare in earlier outbreaks [82]. These clinical findings may help distinguish cases of chikungunya from dengue when rapid diagnostics are not available.

There were slightly more female patients in this cohort. Previous reports have described the transmission dynamics of chikungunya in women when confined to their households [83]. In this cohort, there were no children with chikungunya infection. Disproportional chikungunya infection in adults compared to children has also been previously described [84]. The median (IQR) age of patients in this study was 47 (35.7–55) years, which is consistent with a previous study in Thailand, where the middle-aged group was predominantly affected [4]. Comorbidities were present in 11.5% of the patients but were not associated with increased risk, as observed by others [85]. Fever was present in 76.9% of patients at presentation, and the median (IQR) body temperature was 38 (37.7–39) °C in our patients, similar to that observed previously [14]. Arthralgia is considered a hallmark of chikungunya infection. Arthralgia was the predominant finding in our cohort, with a prevalence up to 92.3%. Similar frequencies of arthralgia have been reported by earlier studies [8,14,86]. Our analysis found that female patients who presented very early during infection, within 1 to 2 days following the onset of symptoms, manifested arthralgia significantly more than male patients. Female sex has been reported previously as an independent risk factor for the development of arthralgia [87,88].

We also analyzed arthralgia by looking at the joints affected. Large joint involvement was significantly more common in female patients who presented within 1 to 2 days than in male patients. Previous reports have found large joint involvement to be more common than small joint involvement, but have not found discrepancies by sex in the involvement of large joints [89]. We also observed higher viremia to be significantly associated with the development of arthralgia. Others have described a high viral load being associated with clinical manifestations during the acute phase of chikungunya in children [90]. A higher viral load has also been associated with the increased expression of pro-inflammatory cytokines such as IL-6 and IL-8 in chikungunya infections [91]. We observed that there was a positive correlation between the increments of circulating neutrophils and viremia. Researchers have demonstrated that neutrophils initiate interferon expression and have used neutrophil extracellular traps to control chikungunya infection [92,93]. Earlier reports suggested that the mean neutrophil counts are slightly higher in chikungunya infection than in dengue and dengue hemorrhagic fever [39]. We also observed higher neutrophil count in chikungunya patients than in dengue patients (Supplementary Table S5).

The hematological profile in chikungunya during the acute phase is consistent with a viral etiology described previously [88]. In severe cases, as well as in atypical cases, leukocytosis can be observed [94]. We found that female patients who presented on the 3rd and 4th day of illness had a decreased leukocyte count compared to males. Further, we also detected a positive correlation between viral load with leukocyte and neutrophil levels. Our analysis revealed an increase in circulating neutrophils, and lymphocytes with the increase of circulating leukocytes. Similarly, there was a significantly decrease in the circulating lymphocytes in the group with increased viremia and an increase of neutrophils with higher viremia. In addition to this, we also observed in our analysis that in patients with a higher viral load, both hemoglobin and hematocrit levels were lower compared to those with low viremia. Limitations of the present study included the small sample number, lack of longitudinal data and inflammation makers such as C-reactive proteins or fibrinogen, and lack of an intensity score or the number of joints involved or an analogical visual pain scale. Further analysis with larger numbers of patients during infection is warranted.

With the lack of approved vaccines and antiviral agents against the CHIKV, preventive and control measures are crucial especially when patients are febrile and presenting with arthralgia. Such measures include mosquito bite prevention, adulticiding, source reduction and social sensitization during outbreaks.

## 5. Conclusions

The chikungunya virus has resurged in Thailand, and it is challenging for clinicians to discriminate chikungunya infection from other circulating arboviruses in endemic regions. Arthralgia was a predominant clinical finding of chikungunya infection, and higher viremia correlates with arthralgia.

## Figures and Tables

**Table 1:** Clinical and hematological profiles of the study cohort (*n* = 26).

Clinical Manifestations	Group A			Group B		
	Female = 8	Male = 5	*p* Value	Female = 6	Male = 7	*p* Value
Fever	7 (87.5)	5 (100)	0.411	5 (83.3)	3 (42.9)	0.135
Arthralgia	8 (100)	3 (60)	**0.** **052**	6 (100)	7 (100)	NA
Large joints	8 (100)	3 (60)	**0.** **052**	4 (66.7)	6 (85.7)	0.416
Small joints	7 (87.5)	3 (60)	0.252	5 (83.3)	6 (85.7)	0.612
Both joints	7 (87.5)	3 (60)	0.252	4 (66.7)	4 (57.4)	0.725
Arthritis	4 (50)	1 (20)	0.279	3 (50)	4 (57)	0.797
Myalgia	4 (50)	3 (60)	0.725	4 (66.7)	5 (71.4)	0.853
Rash	3 (37.5)	2 (40)	0.928	2 (33.3)	5 (71.4)	0.170
Pruritus	1 (12.5)	2 (40)	0.252	2 (33.3)	2 (28.6)	0.853
Headache	3 (37.5)	0	0.118	0	1(14.3)	0.335
Conjunctivitis	0	1 (20)	0.188	2 (33.3)	0	0.097
Diarrhea	0	0	NA	0	1 (28.6)	0.155
**Hematological Profile**						
Leukocytes/µL	5050 (3157–5975)	5100 (3850–6750)	0.884	3400 (2950–4270)	4900 (4500–7400)	**0.** **010**
Lymphocytes/µL	784 (419–1064	969 (812–1435)	0.188	560 (307–735)	1215 (735–1323)	**0.** **015**
Neutrophils/µL	3632 (1419–4899)	3009 (1648–4997)	0.770	2086 (1630–3469)	3234 (2597–5254)	0.086
Eosinophils/µL	26 (0–88)	4.1 (0–112)	0.878	25 (0–88)	0 (0–98)	0.351
Monocytes/µL	131 (58–250)	160 (76–282)	0.509	116 (105–208)	148 (98–243	0.568
Basophils/µL	0 (0–14)	4.1 (0–41.7)	0.317	0 (0–7.18)	0 (0–45)	0.499
Atypical lymphocytes/µL	68 (0–177)	204 (27.5–666)	0.266	70 (0–234)	148 (135–296)	0.153
Bands/µL	183 (19–326))	123 (27–557)	0.768	127 (24–288)	148 (0–441)	1.000
Hemoglobin g/dL	13.1 (12.0–13.8)	13.6 (13.1–16.3)	0.107	13.7 (12.9–14)	14.8 (13.7–14.9)	0.073
Hematocrit %	39.4 (37.8–43.2)	42 (39.3–48.6)	0.271	41.6 (38.3–42.7)	44.6 (41–45.3)	0.063
Platelets 10^3^/µL	189 (142–290)	161 (128–175)	0.188	219 (128–237)	182 (155–246)	0.668
Viral load copies/mL	1.0 × 10^5^ (9.8 × 10^3^–6.2 × 10^5^)	4.7 × 10^3^ (6.5 × 10^2^–5.2 × 10^5^)	0.380	2.9 × 10^5^ (1.1 × 10^5^–5.6 × 10^5^)	8.0 × 10^4^ (1.5 × 10^2^–1.8 × 10^5^)	0.153

Frequencies of clinical manifestations are presented as actual numbers and percentages, while the hematological profiles are presented as medians and IQR. Group A: patients who presented within 1 to 2 days following the onset of symptoms. Group B: patients presented on the 3rd and 4th day of illness. Atypical lymphocytes are lymphocytes observed to be large with varying morphology, similar to those seen in infectious mononucleosis.

**Table 2:** Clinical and hematological profiles based on the level of viremia (*n* = 26).

Viral Load	≥10,000 Copies/mL (*n* = 17)	<10,000 Copies/mL (*n* = 9)	*p* Value
Fever	16 (94.1)	4 (44.4)	**0.** **004**
Arthralgia	17 (100)	7 (77.8)	**0.** **043**
Large joints	14 (82.4)	7 (77.8)	0.778
Small joints	15 (88.2)	5 (55.6)	0.060
Both joints	13 (76.5)	5 (55.6)	0.272
Arthritis	7 (41.2)	5 (55.5)	0.484
Myalgia	12 (70.6)	4 (44.4)	0.192
Rash	9 (52.9)	3 (33.3)	0.340
Pruritus	4 (23.5)	3 (33.3)	0.592
Headache	4 (23.5)	0	0.114
Conjunctivitis	3 (17.6)	0	0.180
Diarrhea	1 (5.9)	1 (11.1)	0.634
Leukocytes/µL	4900 (3650–5850)	4100 (3100–5000)	0.195
Lymphocytes/µL	671 (440–812)	1323 (996–1508)	**0.** **000**
Neutrophils/µL	3469 (2727–4797)	1656 (1155–3121)	**0.** **010**
Eosinophils/µL	0 (0–72)	4.1 (0–121)	0.387
Basophils/µL	135 (102–232)	150 (77–282)	0.808
Monocytes/µL	0 (0–24)	0 (0–18.25)	0.974
Atypical lymphocytes/µL	61 (0–188)	192 (111–414)	0.083
Bands/µL	162 (82–396)	76 (0–166)	0.116
Hemoglobin g/dL	13.6 (12.8–14.1)	14.2 (13.4–15.8)	0.089
Hematocrit %	41.2 (38.2–43.5)	44.6 (40.5–47.4)	**0.** **055**
Platelets 10^3^/µL	178 (140–219)	226 (150–270)	0.319

Frequencies of clinical manifestations are presented as actual numbers and percentages, while hematological profiles are presented as medians and IQR. Atypical lymphocytes are lymphocytes observed to be large with varying morphology, similar to those seen in infectious mononucleosis.

## Data Availability

The data presented in this study are available on request from the corresponding author. The data are not publicly available due to privacy of study participants.

## Supplementary Materials

The following are available online at https://www.mdpi.com/2414-6366/6/1/12/s1, Figure S1: The distribution of white blood cells and hematocrit percentages with respect to the degree of viremia. (a) Lymphocytes vs. viremia, (b) neutrophils vs. viremia, (c) hematocrit vs. viremia, Figure S2: Correlation analysis of leukocytes with other blood cell indices. (a) Leukocytes vs. neutrophils, (b) leukocytes vs. lymphocytes, (c) leukocytes vs. platelets, Figure S3: Correlation analysis of cycle threshold with blood cell indices. (a) Cycle threshold vs. hematocrit, (b) cycle threshold vs. hemoglobin, and (c) cycle threshold vs. neutrophils, Figure S4: Distribution of identified clusters of closely related strains of chikungunya virus within Bangkok. (Markers in the colors orange, blue, and green represent the clusters and the numbers represent the strain identification number, as shown in Table S3), Table S1: The demographic data, Table S2: The clinical data between those who presented within 1 to 2 days after the onset of symptoms (Group A) and 3 to 4 (Group B), Table S3: Levels of chikungunya virus in sera and accession numbers of chikungunya virus genome sequences analyzed in the present study, Table S4: Characteristic amino acid residues of chikungunya viruses analyzed in the present study, Table S5: Comparison of hematological and clinical profile between chikungunya and dengue patients.
