# Supplementary material for: Chikungunya Manifestations and Viremia in Patients Who Presented to the Fever Clinic at Bangkok Hospital for Tropical Diseases during the 2019 Outbreak in Thailand
Source: Trop Med Infect Dis. 2021 Jan 21;6(1):12. doi: 10.3390/tropicalmed6010012 (PMC7924391; doi:10.3390/tropicalmed6010012)
Supplement: Supplementary file 1 [file tropicalmed-06-00012-s001.pdf]

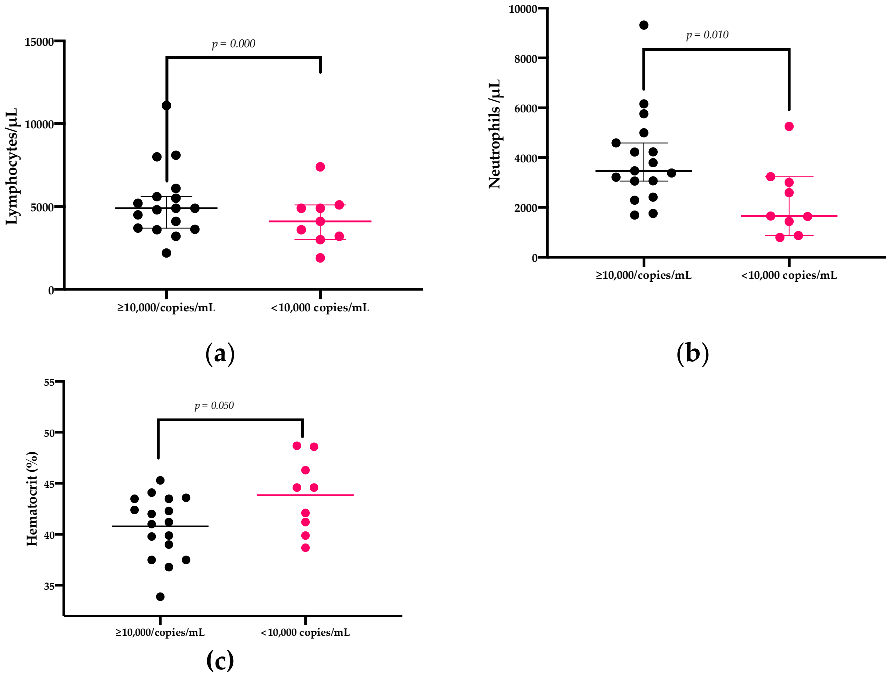

Figure S1. The distribution of white blood cells and hematocrit percentages with respect to the degree of viremia. (**a**) Lymphocytes vs. viremia, (**b**) Neutrophils vs. viremia, (**c**) Hematocrit vs. viremia.

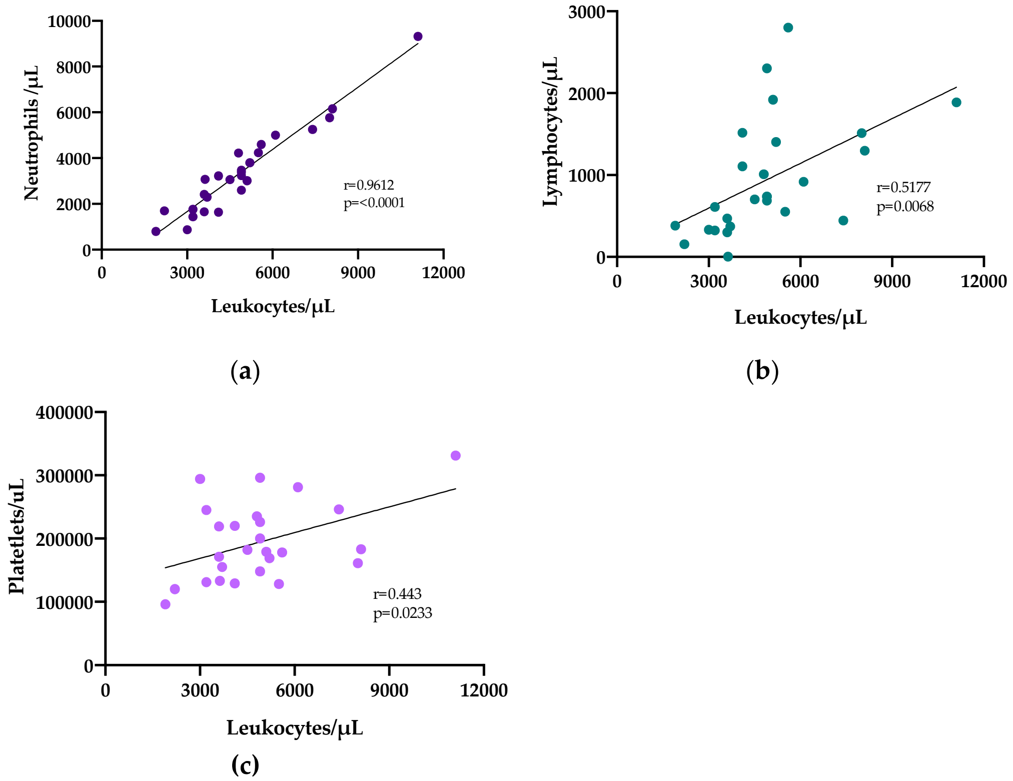

Figure S2. Correlation analysis of leukocytes with other blood cell indices. (**a**) Leukocytes vs. neutrophils, (**b**) Leukocytes vs. lymphocytes, (**c**) Leukocytes vs. platelets.

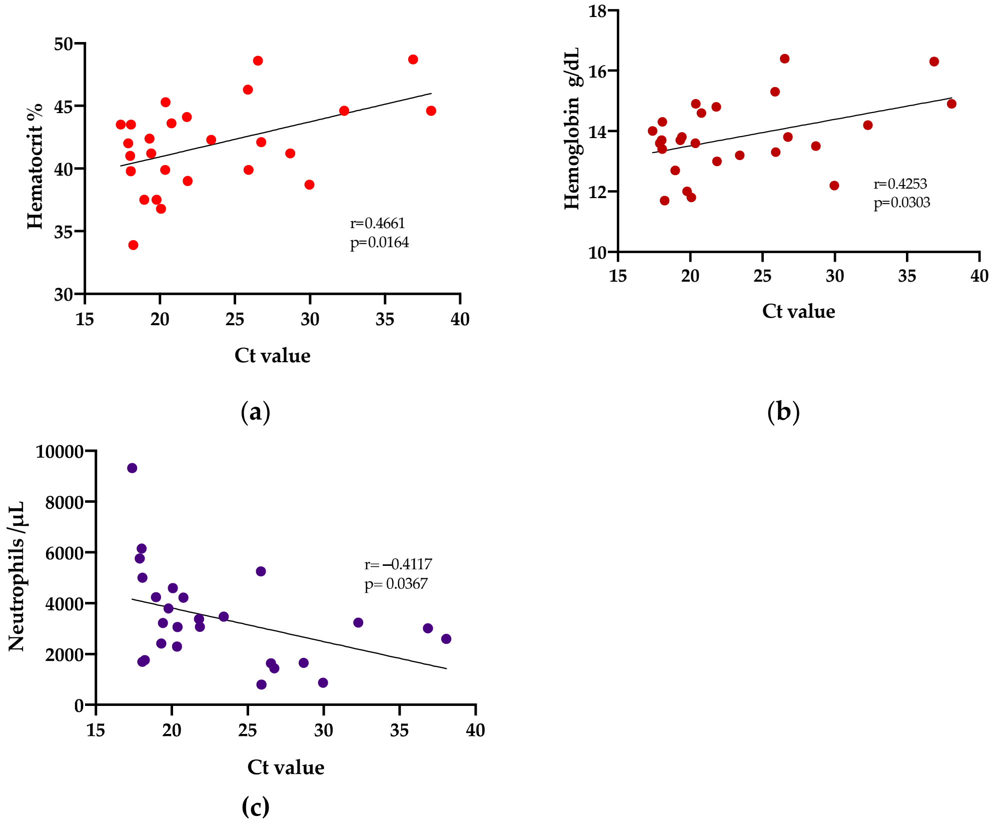

Figure S3. Correlation analysis of cycle threshold with blood cell indices. (**a**) Cycle threshold vs. hematocrit, (**b**) Cycle threshold vs. hemoglobin, and (**c**) Cycle threshold vs. neutrophils.

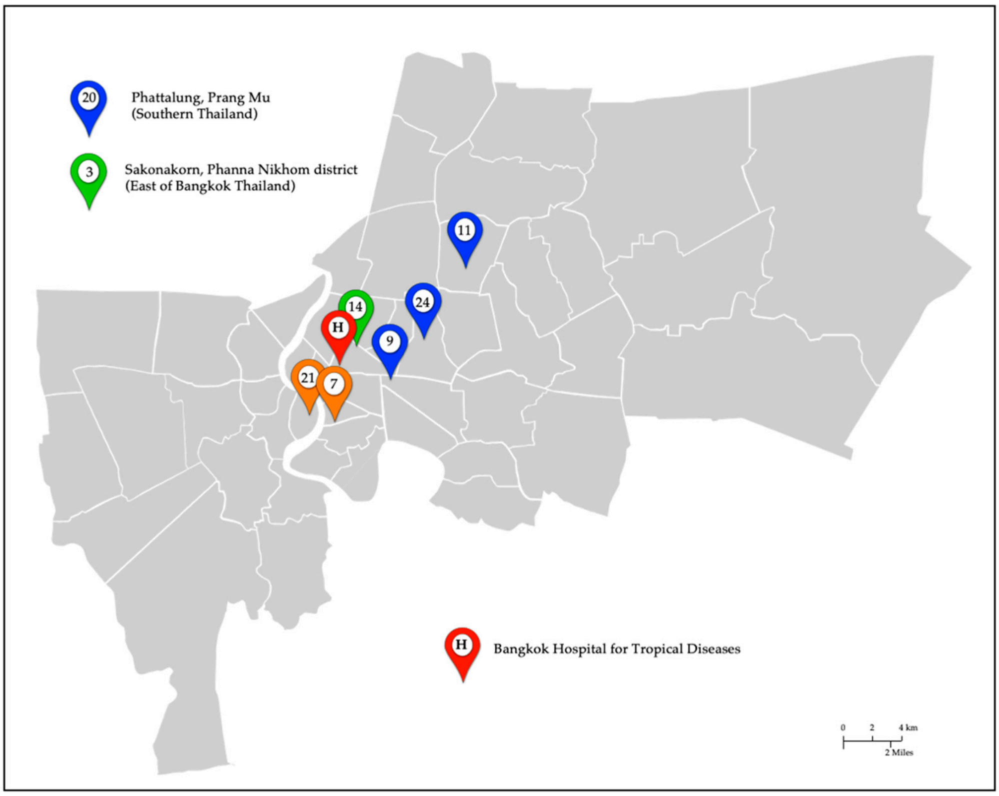

Figure S4. Distribution of identified clusters of closely related strains of chikungunya virus within Bangkok. Markers in the colors orange, blue, green represents the clusters identified by a phylogenetic analysis of Figure 2 and the numbers represent the last two digits of the strain identification number as shown in in Table S3.

Table S1.

**Demographic and clinical data**	**Female=14**	**Male=12**	p value
Age, years	48.5 (31.5–58.7)	45 (37.5–50.7)	0.520
Thai national	13 (93)	12 (92)	0.910
Foreigner	1 (7.1)	1 (8.3)	
Comorbidity	1 (7.1)	2 (17%)	0.449
Day of presentation, days	2 (2–3)	3 (2–3)	0.380
Viral load, copies/mL	$2.0{\times}10^5$ ($1.5{\times}10^4$ – $5.6{\times}10^6$)	$4.3{\times}10^4$ (443 – $3.7{\times}10^5$)	0.157
Temperature, °C	38.25 (37.95–39)	38.25 (37–39.18)	0.706
Fever	12 (86)	8 (67)	0.250
Arthralgia	14 (100)	10 (83)	0.112
Arthritis	7 (50)	5 (42)	0.671
Myalgia	8 (57)	8 (67)	0.619
Rash	5 (35)	7 (58)	0.249
Pruritus	3 (21)	4 (33)	0.495
Headache	3 (21)	1 (8)	0.356
Conjunctivitis	2 (14)	1 (8)	0.636
Diarrhea	0	2 (17)	0.112
Leukocytes/μL	3865 (3150–5300)	4900 (4200–6925)	0.094
Lymphocytes/μL	655 (336–950)	1092 (755–1345)	**0.012**
Neutrophils/μL	3142 (1630–4316)	3147 (2369–4999)	0.572
Eosinophils/μL	23.35 (0–84.98)	0 (0–93.5)	0.582
Monocytes/μL	117.5 (87.25–231.5))	156.5 (107.25–268.65)	0.440
Basophils/μL	0 (0–4.9)	0 (0–41.85)	0.245
Atypical lymphocytes/μL	68.5 (0–183)	176 (75–473)	**0.052**
Bands/μL	150 (24–312)	135 (13–430)	0.856
Hemoglobin g/dL	13.35 (12.15–13.85)	14.5 (13.6–15.2)	**0.013**
Hematocrit, %	40.55 (38.4–42.68)	44.35 (41.05–46.05)	**0.019**
Platelets 10^3/μL	209 (132–254)	175 (149–215)	0.537

Table S2.

**Clinical data**	**Group A**	**Group B**	p value
Fever	12 (92.3)	8 (61.5)	0.063
Arthralgia	11 (84.6)	13 (100)	0.141
Large joints	11 (84.6)	10 (76.9)	0.619
Small joints	10 (76.9)	10 (76.9)	1.000
Both joints	10 (76.9)	8 (61.5)	0.395
Arthritis	5 (38.5)	7 (53.8)	0.431
Myalgia	7 (53.8)	9 (69.2)	0.420
Rash	5 (38.5)	7 (53.8)	0.431
Pruritus	3 (23.1)	4 (30.8)	0.658
Headache	3 (23.1)	1 (7.7)	0.277
Conjunctivitis	1 (7.7)	2 (15.4)	0.539
Diarrhea	0	2 (15.4)	0.414
Leukocytes/μL	5100 (3615–5850)	4500 (3400–4900)	0.329
Lymphocytes/μL	893 (673–1231)	740 (603–1323)	0.442
Neutrophils/μL	3469 (1648–4797)	3060 (2027–3802)	0.663
Eosinophils/μL	4.1 (0–86)	0 (0–81)	0.722
Monocytes/μL	153 (59–268)	135 (103–219)	0.959
Basophils/μL	0 (0–26)	0 (0–14)	0.510

Atypical lymphocytes/μL	76 (0–206)	147 (46.5–269)	0.624
Bands/μL	156 (27–362)	144 (16–316)	0.796
Hemoglobin g/dL	13.3 (12.45–14.15)	13.8 (13.65–14.85)	0.101
Hematocrit %	41.2 (38.1–43.5)	42.4 (40.45–44.6)	0.270
Platelets 10^3/μL	171 (131–240)	219 (151–240)	0.369
Viral load copies/mL	5.0×10^4 (3.0×10^3–5.2×10^5)	1.8×10^5 (5.9×10^3–4.1×10^5)	0.837

Frequencies of clinical manifestations are presented as actual numbers and percentages, while hematological profiles are presented as medians and IQR. Atypical lymphocytes are lymphocytes observed to be large with varying morphology, similar to those seen in infectious mononucleosis.

Table S3.

**Strain**	**Collection date**	**Sex**	**Age (years)**	**Ct**	**Viral load (copies/mL)**	**Accession No.**
THBKK19-01	October 2019	F	40	26.76	4.73E+03	LC598202
THBKK19-02	October 2019	M	50	36.87	9.99E+00	Undetermined
THBKK19-03	October 2019	M	43	28.69	1.31E+03	LC598203
THBKK19-04	October 2019	F	24	19.79	2.66E+05	LC580256[b]
THBKK19-05	October 2019	M	44	38.08	4.86E+00	Undetermined
THBKK19-06	October 2019	M	55	20.38	1.87E+05	LC580257[b]
THBKK19-07	October 2019	F	61	29.47	8.24E+02	LC598204
THBKK19-08	October 2019	F	49	19.43	3.29E+05	LC580258[b]
THBKK19-09	October 2019	M	17	26.54	4.73E+03	LC580259[b]
THBKK19-10	October 2019	M	37	32.28	1.54E+02	Undetermined
THBKK19-11	October 2019	M	28	21.8	8.00E+04	LC580260[b]
THBKK19-12	October 2019	F	58	25.91	6.90E+03	LC580261[b]
THBKK19-13	October 2019	M	39	20.36	1.89E+05	LC580262[b]
THBKK19-14	October 2019	F	64	18.06	7.48E+05	LC580263[b]
THBKK19-15	October 2019	F	32	20.77	1.48E+05	LC580264[b]
THBKK19-16	October 2019	M	55	25.87	7.08E+03	LC580265[b]
THBKK19-17	October 2019	F	61	20.08	1.55E+05	LC580266[b]
THBKK19-18	October 2019	F	50	29.97	6.14E+02	LC598205
THBKK19-19	October 2019	F	67	18.08	7.39E+05	LC580267[b]
THBKK19-20	October 2019	M	46	18.97	4.35E+05	LC580268[b]
THBKK19-21	October 2019	F	39	17.39	1.87E+06	LC580269[b]
THBKK19-22	October 2019	F	25	18.23	5.03E+05	LC598206
THBKK19-23	October 2019	M	48	17.9	6.21E+05	LC598207
THBKK19-24	October 2019	F	30	23.42	1.87E+04	LC598208
THBKK19-25	October 2019	F	55	18.02	5.75E+05	LC598209
THBKK19-26	October 2019	F	48	19.31	2.53E+05	LC598210

Table S4.

**Position**	**Structural polyprotein**						
	73	530	546	589	1020	1035	1126
	**capsid**		**E2**			**E1**	
	73	205	221	264	211	226	317
Amino acid residue	R	S	K	A	E	A	V

Table S5.

**Day 2 of illness**	**Chikungunya=7**	**Dengue=7**	p value
Fever	6 (85.7)	7 (100)	0.299
Headache	2 (28.6)	7 (100)	**0.005**
Myalgia or arthralgia	7 (100)	6 (85.7)	0.299
Rash	3 (42.9)	4 (57.1)	0.593
Bleeding	0	0	NA

Leukocytes/μL	5600 (3600–8000)	3500 (2300–5400)	0.085
Lymphocytes/μL	800 (336–1110)	560 (345–822)	0.482
Neutrophils/μL	4592 (3042–5760	2590 (1587–3450)	0.180
Atypical Lymphocytes/μL	61 (0–180)	0 (0–46)	0.096
Hemoglobin g/dL	13 (12.2–14)	15.4 (13.8–15.6)	**0.048**
Hematocrit %	39 (37.5–43.5)	44.5 (40.4–45.3)	0.141
Platelets 10^3/μL	178 (133-294)	154 (68-212)	0.142
**Day 3 of illness**	**Chikungunya=15**	**Dengue=22**	
Fever	12 (80)	22 (100)	**0.029**
Headache	2 (13.1)	20 (90.9)	**0.000**
Myalgia or arthralgia	14 (93.3)	19 (86.4)	0.503
Rash	7 (46.7)	12 (54.5)	0.638
Bleeding	0	5 (22.7)	**0.047**
Leukocytes/μL	4100 (3200–4900)	3000 (2380–4200)	0.068
Lymphocytes/μL	735 (576–1024)	619 (421–997)	0.496
Neutrophils/μL	2419 (1694–3381)	1659 (1214–2436)	**0.041**
Atypical Lymphocytes/μL	148 (49–243)	190 (72–394)	0..395
Hemoglobin g/dL	13.8 (13.4–14.9)	13.9 (12.3–15.0)	0.699
Hematocrit %	42.1 (39.9–44.6)	41.1 (37.4–43.5)	0.240
Platelets 10^3/μL	179 (131–220)	87 (62.2–127)	**0.000**
**Day 4 of illness**	**Chikungunya=3**	**Dengue=43**	
Fever	1 (33.3)	43 (100)	**0.000**
Headache	0	41 (95.3)	**0.000**
Myalgia or arthralgia	3 (100)	33 (76.7)	0.345
Rash	1 (33.3)	21 (48.8)	0.603
Bleeding	0	12 (27.9)	0.287
Leukocytes, 10^3/μL	4900 (3600)	2900 (2300–4200)	0.071
Lymphocytes, %	1353 (1323)	744 (456–1248)	0.065
Neutrophils, %	3234 (1656)	1406 (1100–1974)	**0.039**
Atypical Lymphocytes, %	147 (0)	330 (140–720)	0.175
Hemoglobin, %	14.2 (13.5)	15 (13.3–16)	0.533
Hematocrit, %	44.6 (41.2)	44 (40.3–47.1)	0.965
Platelets, 10^3/μL	226 (171)	62.5 (30–87.2)	0.000

The data are presented as medians and IQR. Atypical lymphocytes are lymphocytes observed to be large with varying morphology, similar to those seen in infectious mononucleosis.
